# Improvement in the potency of a N^1^-methylpseudouridine-modified self-amplifying RNA through mutations in the RNA-dependent RNA polymerase

**DOI:** 10.1016/j.jbc.2025.110487

**Published:** 2025-07-17

**Authors:** Verónica Quintana, Josefina Caillava, Laura A. Byk, Juan A. Mondotte, Leandro Battini, Prutha Tarte, Marcelo M. Samsa, Claudia V. Filomatori, Diego E. Alvarez

**Affiliations:** 1 Instituto de Investigaciones Biotecnológicas, Universidad Nacional de San Martín-CONICET, Argentina; 2 GreenLight Biosciences Inc, Lexington, Massachusetts, USA

**Keywords:** RNA vaccines, modified nucleotides;, alpha virus, polymerase fidelity

## Abstract

mRNA technology has widely incorporated modified nucleotides to temper innate immune activation by foreign RNA and achieve high levels of heterologous protein expression. However, the incorporation of modified nucleotides has had limited application to self-amplifying (sa) RNAs, as it impeded heterologous protein expression. Here, we used a reporter replicon of the attenuated TC-83 strain of Venezuelan equine encephalitis virus (VEEV) to investigate the impact of modified nucleotide incorporation on the replication capacity of the saRNA in transfected cells. ψ and m^1^ψ-modified molecules exhibited a profound defect in RNA synthesis compared to unmodified saRNA. Interestingly, the RNA synthesis levels of m^5^C-modified RNAs were similar to unmodified molecules, positioning m^5^C as a promising candidate for saRNA modification. Moreover, we found that saRNAs carrying mutations associated with resistance to nucleotide analog polymerase inhibitors partially overcame the negative impact of m^1^ψ-modified nucleotide incorporation on RNA synthesis. Overall, we uncovered a previously unappreciated link between polymerase fidelity and saRNA amplification.

RNA technologies involve the *in vitro* transcription of synthetic RNA molecules that, upon delivery into a host cell, serve as messenger RNAs (mRNAs) to achieve native expression of a heterologous protein. The successful development of mRNA technologies has relied on different strategies aimed at increasing the yield of heterologous protein expression (1). First, capping with analogs that mimic endogenous mRNA molecules. Second, optimization of 5′ and 3′ untranslated regions (UTRs), and poly(A) tail to enhance translational efficiency. Third, incorporation of modified nucleotide analogs, including 5-methylcytidine (m^5^C), N6-methyladenosine (m^6^A), 5-methyluridine (m^5^U), 2-thiouridine (s^2^U), N^1^-methylpseudouridine (m^1^ψ) or pseudouridine (ψ), to reduce immune activation in transfected cells (2) and increase the translational capacity of modified mRNAs (3). In turn, self-amplifying RNAs (saRNAs) can replicate in the cell cytoplasm, generating multiple copies of translatable RNAs that assure high yields of heterologous proteins (4, 5).

saRNAs are derived from the genomes of positive-strand RNA viruses. The most usual design follows the organization of alphavirus genomes, preserving viral 5′ and 3′UTRs (6). A first open reading frame (ORF) encodes the replicase complex, which consists of non-structural proteins 1 to 4 (nsP1–4): nsP1 anchors the complex to the plasma membrane and is required for viral RNA capping, nsP2 functions as an RNA helicase and viral protease, nsP3 serves as a scaffold for the recruitment of host factors, and nsP4 acts as the RNA-dependent-RNA-polymerase (RdRp). A second ORF, under the control of a subgenomic promoter, encodes a heterologous protein in place of the original viral structural proteins. Upon delivery into the cell cytoplasm, saRNA molecules are first translated to produce the nsP1–4 proteins that assemble into the replicase complex. The complex uses the input RNA molecule as a template to synthesize a complementary RNA copy, which in turn serves as a template for the transcription of full-length and subgenomic RNAs. Therefore, the heterologous protein is translated only from the subgenomic RNA, once the input RNA has been transcribed and amplification has occurred within the first few hours through subsequent rounds of replication.

Innate immune activation is a major issue in saRNA technology. In addition to the high immunogenicity of the synthetic molecule, cells elicit a response against replication intermediates and viral antigens (7). Since antigen expression requires the initial translation of replicase components, the immune response mounted upon saRNA delivery into the host cell directly impacts the yield of antigen expression. Noteworthy, unlike mRNA vaccines, saRNAs do not accept modified nucleotides as a strategy to reduce immunogenicity (8, 9).

Here, we investigated the impact of incorporating modified nucleotides into a saRNA molecule derived from the TC-83 attenuated strain of VEEV. We observed that while modified saRNAs are efficiently translated, they exhibit impaired RNA synthesis in cell culture. Therefore, optimizing replicase components offers an attractive alternative to enhance payload expression from saRNA. To engineer a viral replicase capable of tolerating modified nucleotides, we introduced substitutions in nsp4 that have been reported to alter fidelity (10, 11). Importantly, we found that replicons carrying mutations associated with a high-fidelity RdRp phenotype replicated m^1^ψ-modified saRNAs at higher levels and displayed higher infectivity than wild type replicons. Altogether, our findings establish a novel link between polymerase fidelity and its ability to utilize modified saRNAs as templates for RNA synthesis.

## Results

### Assessment of translation and replication ability of VEEV-based saRNAs incorporating modified nucleotides

**Figure 1:**
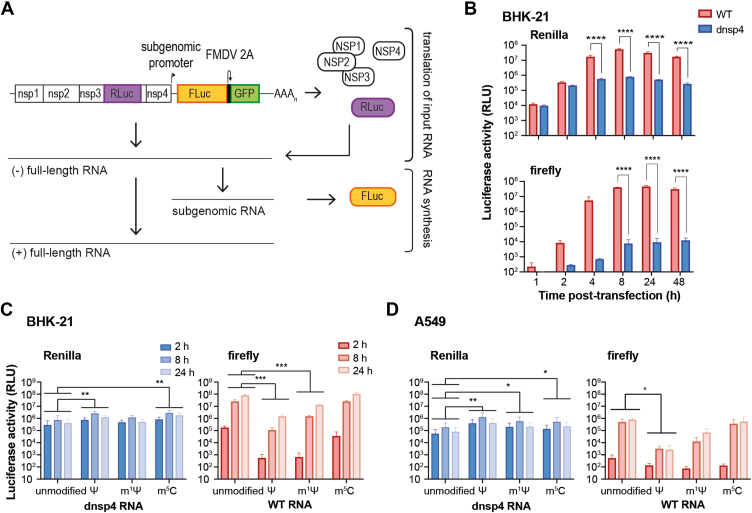
**Uridine analogs impair the self-amplifying ability of a VEEV-based replicon RNA.***A*, schematic representation of a VEEV TC-83 -based reporter replicon. Renilla luciferase sequence (RLuc) was inserted in the hypervariable region of nsp3. Firefly luciferase (FLuc) and GFP sequences separated by FMDV 2A autoprotease cleavage sequence were inserted under the control of the subgenomic promoter within the second ORF. *B*, bar graph showing Renilla (*top*) and firefly (*bottom*) luciferase activity as a function of time after transfection of BHK-21 cells with 80 ng RNA of VEEV replicon (WT, *red bars*) or catalytically inactive nsP4 mutant (dnsp4, *blue bars*). Statistical analysis was performed using two-way ANOVA, followed by Sidak’s test to compare means at each time point. *C* and *D*, bar graph displaying Renilla luciferase activity of dnsp4 construct (*C* and *D*, *left*) and firefly luciferase activity of WT (*C* and *D*, *right*) at 2, 8 and 24 h post-transfection into BHK-21 (*C*) or A549 (*D*) cells of VEEV TC-83 replicon saRNA transcribed *in vitro* with unmodified nucleotides or with ψ, m1ψ or m^5^C. Statistical analysis of Renilla luciferase levels compared the geometric means of the ratios between unmodified and modified RNAs and was performed using repeated measures one-way ANOVA of log-transformed values followed by Dunnett’s multiple comparison test. For firefly luciferase levels, the main effect of modified nucleotides vs. unmodified was compared using two-way ANOVA followed by Dunnett’s test. ns *p* > 0.05; ∗*p* ≤ 0.05; ∗∗*p* ≤ 0.01; ∗∗∗*p* ≤ 0.001; ∗∗∗∗*p* ≤ 0.0001. Error bars indicate the standard deviation. Two independent experiments with two replicates per condition were performed.

We designed a reporter VEEV-based replicon carrying the Renilla luciferase gene inserted into the first ORF, and the firefly luciferase gene followed by the 2A autoprotease from foot and mouth disease virus (FMDV) and GFP genes in place of the viral structural proteins within the second ORF (Fig. 1*A*). In this construct, Renilla luciferase is translated directly from the input saRNA upon transfection, serving as a readout of genomic translation. In turn, firefly luciferase is translated from the subgenomic RNA, which is produced by the viral replicase following an initial round of translation and RNA synthesis. In addition to the wild-type (WT) replicon, we generated a mutant replicon (dnsp4), bearing a mutation in the nsp4 RdRp catalytic site that turns the polymerase inactive (12).

Alphavirus-derived modified saRNAs reported to be non-functional *in vivo*, were enzymatically capped after *in vitro* transcription to create a cap 0 structure (m^7^GpppAU) (8). Since a cap 1 structure (m^7^GpppAmU) reduces immune detection compared to cap 0, we cotranscriptionally capped *in vitro* transcribed RNAs with the CleanCap AU that features a cap 1. First, WT and dnsp4 saRNAs were transfected into BHK-21 cells to characterize the expression kinetics of the luciferase reporter genes (Fig. 1*B*). Renilla luciferase activity was detected as early as 1 hour after transfection, displaying similar levels for the WT and dnsp4 constructs. At later time points, Renilla activity increased for both WT and the dnsp4 constructs, but as of 4 hours post-transfection, a significant difference was observed, reflecting genomic RNA amplification by an active replicase complex (Fig. 1*B*, top). In turn, firefly luciferase activity for the WT replicon was detectable at low levels above background at 1 hour and became readily detectable by 2 hours post-transfection (Fig. 1*B*, bottom). Then, it increased exponentially, reaching a maximum at 8 hours. In contrast, firefly luciferase levels for the dnsp4 construct showed only a marginal increase throughout the time course and remained approximately 1000-fold below WT levels, suggesting that the mutated nsP4 retains leaky RdRp activity (Fig. 1*B*, bottom). Therefore, our reporter system allowed monitoring input saRNA translation by means of dnsp4 Renilla luciferase activity at early time points, and RNA synthesis through firefly luciferase activity of the WT construct.

To investigate the impact of nucleotide analog incorporation on saRNA amplification, we synthesized unmodified or modified saRNAs for both WT and dnsp4 mutant replicons. Clinically approved mRNA vaccines use m^1^ψ. In turn, an m^5^C-modified saRNA was shown to achieve higher expression levels than the unmodified RNA (13). Then, we performed *in vitro* transcriptions of the reporter saRNA using 100% ψ or 100% m^1^ψ in place of uridine, or 100% m^5^C in place of cytidine. These molecules were then transfected into BHK-21 cells, and Renilla luciferase activity was monitored at 2-, 8-, and 24-h post-transfection (Fig. 1*C*, left). Both ψ- and m^5^C-modified saRNAs displayed overall 3-fold higher luciferase levels compared to the unmodified saRNA across the time course, while m^1^ψ-modified saRNA yielded similar luciferase levels to the unmodified saRNA. Using firefly luciferase levels to assess saRNA replication in the WT background, we observed that incorporation of ψ and m^1^ψ resulted in a 100-fold decrease in luciferase levels that was evident as early as 2 h post-transfection (Fig. 1*C*, right). Remarkably, m^5^C-saRNA showed luciferase levels comparable to the unmodified saRNA (Fig. 1*C*, right). These results indicate that saRNAs transcribed with 100% ψ or with 100% m^5^C have an advantage in translation of the input RNA, while modification with ψ or with m^1^ψ analogs impairs replicase function.

It has been widely reported that the incorporation of nucleotide analogs diminishes immune sensing of foreign RNAs (3, 14). Since the initial characterization of our reporter constructs was conducted in BHK-21 cells that are deficient in interferon production (15), we hypothesized that modified saRNAs may have an advantage over unmodified saRNAs in cells possessing intact antiviral sensing and interferon signaling pathways. To explore this, we transfected modified or unmodified saRNAs into A549 (Fig. 1*D*). Similar to BHK-21 cells, Renilla luciferase levels in A549 cells indicated increased translation of modified saRNAs, including m^1^ψ transcripts, relative to unmodified saRNA (Fig. 1*D*, left). In turn, firefly luciferase activity indicated that incorporation of ψ and m^1^ψ resulted in overall reduced replication levels, while m^5^C had no apparent effect on RNA replication (Fig. 1*D*, right).

Altogether, similar to mRNAs, the incorporation of modified nucleotides into the saRNA enhances translation of the input saRNA (3, 14). However, ψ- and m^1^ψ-modified saRNAs had reduced self-amplifying ability, whereas m^5^C-modified saRNA displayed levels of self-amplifying capacity comparable to unmodified saRNA.

### Modulation of VEEV RNA-dependent RNA-polymerase activity

Given that our results suggested that the incorporation of modified uridine analogs impaired saRNA replication, we aimed at optimizing replicase function by modulating the RdRp activity of nsp4. It has been previously reported that uridine analogs are incorporated with lower fidelity than unmodified uridine during *in vitro* transcription (16). In addition, synthetic nucleotide analogs used as antiviral agents have been shown to be mutagenic (17). Based on these observations, we speculated that the incorporation of modified analogs would increase the mutation rate due to their mutagenic effects during *in vitro* transcription of the input saRNA and the first round of RNA synthesis by the viral RdRp. Such hypermutated RNAs would push the viral replicon beyond the threshold of viability, explaining the negative impact of modified nucleotide incorporation on replicon RNA amplification. If that were the case, we reasoned that modulating the RdRp fidelity could diminish the error rate during RNA synthesis and mitigate the detrimental effects of nucleotide analog incorporation. For various RNA viruses, growth in the presence of nucleotide analogs has led to the identification of residues in RdRp associated with polymerase fidelity (18, 19). In the case of the related alphavirus chikungunya virus (CHIKV), a mutation at position 483 (C483Y) conferred resistance to ribavirin and was related to a high-fidelity phenotype (10). In addition, CHIKV resistance to favipiravir arose from a substitution at position 291 (K291R) of nsP4 (11).

**Figure 2:**
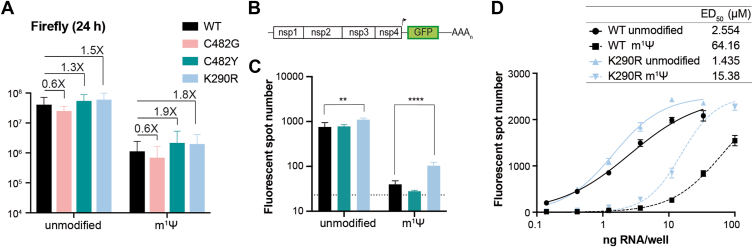
**Mutations associated with increased RdRp fidelity partially rescue the impact of m^1^ψ modification on the self-amplifying ability and the ability to initiate a replication cycle of VEEV TC-83 replicon.***A*, bar graph showing firefly luciferase activity at 24 h post-transfection of BHK-21 cells with 80 ng of unmodified RNA or m^1^ψ-modified RNAs of the WT or the nsp4 mutants C482G (associated with a low fidelity phenotype), and C482Y and K290R (associated with high fidelity phenotypes). *B*, schematic representation of a reporter VEEV TC-83 replicon expressing GFP under the control of the subgenomic promoter in the second ORF. *C*, bar graph showing foci counts in BHK-21 cells transfected with 1 ng of unmodified or m^1^ψ modified saRNA of the WT and the nsp4 C482Y and K290R mutants. GFP-positive foci were numbered 24 h post-transfection in an ImmunoSpot Analyzer, and quantification was used as a measure of RNA transfection efficiency. Statistical analysis was performed using multiple t-tests, followed by the Holm-Sidak method to correct for multiple comparisons. ns *p* > 0.05; ∗∗*p* < 0.01; ∗∗∗∗*p* ≤ 0.001. The data represent the mean of two independent experiments. Error bars indicate the standard deviation. *D*, dose–response curves of infectivity in BHK-21 cells transfected with increasing amounts of unmodified or m1ψ-modified RNAs of the WT or nsp4 K290R mutant. One representative experiment out of three is shown.

To address the impaired replication rate of modified saRNA through modulation of polymerase fidelity, we introduced the mutations associated with CHIKV resistance to ribavirin and favipiravir into the analogous VEEV nsp4 residues (C482Y and K290R, respectively) in the reporter WT saRNA. In addition, we engineered a replicon containing the C482G substitution, which is associated with a low-fidelity phenotype in CHIKV (20). saRNAs were *in vitro* transcribed with unmodified nucleotides or with m^1^ψ and transfected into BHK-21 cells (Fig. 2*A*). Assessment of RNA replication through measurement of firefly luciferase activity at 24 h post-transfection (Fig. 2*A*) showed comparable luciferase levels for all the unmodified saRNAs (Fig. 2*A*, right). In contrast, for m^1^ψ-RNAs, the C482Y and K290R mutants displayed a ∼2-fold increase in firefly luciferase levels, while the low-fidelity C482G mutant displayed similar luciferase levels compared to the WT saRNA (Fig. 2*A*, right).

To evaluate the ability of saRNAs to initiate a replication cycle in a transfected cell, we engineered the K290R mutation into a new reporter VEEV replicon, consisting of a first ORF coding for an uninterrupted sequence of replicase proteins and a second ORF coding for GFP as a payload (Fig. 2*B*). We synthesized the unmodified and modified saRNAs, transfected them into BHK-21 cells, and counted the number of GFP-expressing foci (Fig. 2*C*). Pairwise comparisons between WT and C482Y backgrounds showed similar foci counts for both unmodified saRNAs and m^1^ψ-modified saRNAs. In turn, the unmodified K290R mutant saRNA showed an enhanced ability to initiate a replicative cycle, yielding a 0.5-fold increase in foci counts compared to the unmodified WT saRNA. Noteworthy, for the m^1^ψ-saRNAs, the increase was 2.5-fold. This suggests that the positive effect of the K290R mutation was more pronounced for modified than for unmodified RNAs, implying that tuning the polymerase function could serve as a strategy to overcome the impaired function of nucleotide-modified saRNAs.

To further characterize the phenotype of the K290R mutant, we transfected increasing amounts of saRNA into BHK-21 cells (Fig. 2*D*). After 24 h, we counted the number of foci to estimate the 50% effective dose (ED_50_), defined as the amount of transfected saRNA necessary to reach 50% of the maximum foci number. For the unmodified WT saRNA, the ED_50_ was 2.5 ng, and the incorporation of m^1^ψ increased the ED_50_ about 25-fold (Fig. 2*D*), confirming that m^1^ψ diminishes the number of GFP-expressing cells. The unmodified K290R mutant showed a 2-fold lower ED_50_ than the WT counterpart, indicating that the mutation itself confers an advantage for heterologous protein expression. While the incorporation of m^1^ψ into the K290R saRNA impaired GFP expression, it was remarkable that the ED_50_ increased by 10-fold, which, compared to the 25-fold increase observed for the WT saRNA, suggested that the K290R mutation partially counteracted the negative effect of the nucleotide analogue on protein expression. Noteworthy, the K290R mutant m^1^ψ-saRNA reached maximum foci counts at a plateau similar to the unmodified saRNA, although at higher doses. Thus, we were able to fully overcome the negative effect of nucleotide analogs by combining the K290R mutation with higher doses of saRNA. Altogether, the mutation K290R in nsp4 alleviated the detrimental effect of m^1^ψ-incorporation, suggesting that modulation of RdRp activity could provide a strategy to extend the use of modified nucleotides to saRNA-based therapeutics.

## Discussion

In this study, we investigated the effect of nucleotide analogs on saRNA payload expression. We found that m^1^ψ, ψ and m^5^C incorporation moderately increased RNA translation. While m^1^ψ- and ψ-modified saRNAs negatively affected RNA replication, saRNAs incorporating m^5^C showed replication levels similar to unmodified saRNAs. Importantly, the negative impact of m^1^ψ-modified saRNAs on replication was partially alleviated by point mutations associated with increased viral polymerase fidelity. The incorporation of modified nucleotides is extensively used to improve translation efficiency, enhance stability, and reduce innate immune responses triggered by the delivery of heterologous mRNA (3, 14). However, in the context of saRNA, modified nucleotides appeared to be deleterious to payload expression in animal models. For instance, expression of a human monoclonal antibody against Zika virus, driven from a VEEV TC-83 derived replicon, was undetectable in mice inoculated with the ψ-modified saRNA formulated in lipid nanoparticles (8). Similarly, the induction of an immune response against SARS-CoV-2 was completely abrogated by the incorporation of m^1^ψ into a saRNA expressing the virus spike protein (9). Our findings provide insight into the molecular mechanisms behind the failure of modified saRNAs to express the payload *in vivo*. We identified that the incorporation of modified nucleotides affects the function of the replication complex (Fig. 1, *C* and *D*). Strikingly, ψ- and m^1^ψ-modified saRNAs showed over 100-fold reduction in replication efficiency compared to the unmodified saRNA, a phenomenon also noted with a Semliki Forest virus replicon (21). In contrast, m^5^C did not appear to affect RNA replication. On the other hand, and consistent with previous reports (3), incorporation of ψ, m^1^ψ, or m^5^C resulted in a moderate increase in translation levels of saRNAs (Fig. 1, *C* and *D*). Remarkably, this effect was observed primarily in saRNAs with impaired RdRp activity. As we were able to detect saRNA replication as early as 2 h after transfection, we interpreted that the negative impact of nucleotide incorporation at the stage of RNA synthesis masked the stimulatory effect on translation.

Previous research has explored m^5^C as an alternative modification to m^1^ψ. m^5^C modification has been found to enhance RNA translation efficiency (3) and reduce innate immune responses (2, 14). Immunogenicity studies have demonstrated that m^5^C-modified saRNAs induce decreased activation of human dendritic cells and cells stably expressing TLR3, TLR7, or TLR8 (2). In addition, a recent study reported that the complete substitution of a VEEV replicon with m^5^C is a viable strategy for vaccine development (13). Our data demonstrate that, unlike uridine analogs, m^5^C did not impact the self-amplifying ability of a replicon saRNA (Fig. 1, *C* and *D*). Together with previous studies, these findings underscore the potential of m^5^C-modified saRNA for therapeutic purposes.

Modified nucleotides have mutagenic potential due to altered base-pairing patterns and interactions between modified nucleotides and RNA polymerases during transcription (16, 22). For instance, increased error rates have been reported in the *in vitro* synthesis of RNAs incorporating ψ by T7 RNA polymerase, and to a lesser extent, in RNAs incorporating m^1^ψ. A similar increase in error rates was observed for reverse transcriptases using modified RNAs as templates (16). The saRNA vaccine technology relies on the *in vitro* transcription of an RNA molecule, which then serves as the template for RNA synthesis by the virus replicase upon delivery into target cells. Therefore, the cumulative effect of errors from both *in vitro* transcription and viral RNA synthesis may impair the self-amplifying ability of modified saRNAs, potentially leading to error catastrophe. Our data shows that mutations in the nsp4 RdRp of VEEV TC-83, which were originally selected by passaging the closely related CHIKV in the presence of nucleotide analogs, and are associated with a high-fidelity phenotype, can partially mitigate the impact of uridine analogs incorporation on RNA replication (Fig. 2). Whether nucleotide-modified saRNA templates increase the error rate of the viral replicase warrants experimental demonstration, as well as the assessment of nucleotide misincorporation by nsp4 fidelity variants during minus-strand RNA synthesis. Lys residue at position 290 maps to RdRp motif F and is conserved across polymerases of diverse viruses. This motif interacts with the incoming nucleotide through positively charged residues and has been implicated in fidelity (23, 24). We speculate that the K290R mutation would impact the accurate incorporation of modified bases.

In conclusion, our findings, combined with previous studies, support the incorporation of m^5^C into saRNAs, as it would likely strike an appropriate balance between eliciting immune responses and having minimal impact on the self-amplifying ability of viral replicons. Furthermore, we uncovered an important link between polymerase fidelity and the replication of m^1^ψ-modified self-amplifying RNA molecules. Therefore, engineering the replicase function may serve as a strategy to improve the potency of saRNA platforms and enhance their efficacy in future clinical studies.

## Experimental procedures

### Cells

Cell lines were grown at 37 °C in a 5% CO_2_ atmosphere. BHK-21 cells (ATCC, CCL-10) were grown in α-modified Eagle's minimum essential medium (α-MEM) supplemented with 10% fetal bovine serum (FBS) and penicillin−streptomycin antibiotics. Human lung cell line A549 (ATCC CL-185) was cultured with DMEM supplemented with 10% FBS and antibiotics.

### Constructs

The VEEV-GFP replicon was derived from a plasmid encoding the VEEV TC-83 sequence (GenBank accession number L01443.1) under the control of a T7 promoter by inserting the GFP coding sequence downstream of the subgenomic promoter and upstream of the 3′UTR. The VEEV replicon with the luciferase reporters was constructed by restriction-free cloning of Renilla luciferase coding gene in the hypervariable region of nsp3 (after codon 374), and firefly luciferase coding gene downstream of the subgenomic promoter and upstream of GFP coding gene in VEEV-GFP. nsp4 mutants were generated using a restriction-free approach (25) involving: (i) a first PCR to amplify the insert sequence with designed mutations (Table S1); (ii) a second PCR using the insert sequence as a mega-primer and VEEV replicon plasmid as a template, and (iii) *Dpn*I digestion and transformation of the second PCR product.

### *In vitro* transcription and purification of saRNAs

The VEEV replicon reporter was linearized by restriction digestion with *BspQ*I at the precise 3′ end of the plasmid sequence and purified by a EasyPure PCR Purification Kit (TransGen Biotech). Linearized DNAs were used as templates for *in vitro* transcription of capped RNAs with HiScribe T7 High Yield RNA Synthesis Kit (New England Biolabs) in the presence of CleanCap Reagent AU (TriLink Biotechnologies). For modified saRNAs, UTP was fully substituted with either pseudouridine-5′-triphosphate or N1-Methylpseudouridine-5′-Triphosphate, or CTP was fully substituted with 5-methylcytidine-5′-triphosphate (TriLink Biotechnologies) in the *in vitro* transcription reactions. The reaction mixes were incubated at 37 °C for 2 h. Then, DNA templates were digested with DNase (Invitrogen) at 37 °C for 30 min and the saRNA transcripts were precipitated with lithium chloride overnight at −70 °C. Next, saRNAs were recovered by centrifugation at 12,000 ×*g* for 1 h at 4 °C, washed with 70% ethanol and centrifugation at 12,000 ×*g* for 5 min at 4 °C, and resuspended in nuclease-free water. The quality of the saRNA samples was analyzed by 1% agarose gel electrophoresis. The concentration of saRNA was determined in a Nanodrop spectrophotometer (Thermo Fisher Scientific).

### RNA transfection, measurements of firefly and Renilla Luciferase activities, and counting of GFP-expressing foci

BHK-21 or A549 cells were seeded into 96-well plates at 3.5 × 10^4^ cells per well. After 24 h, *in vitro* transcribed saRNAs were transfected into cultured cells using 0.2 μl of Lipofectamine-2000 (Invitrogen) according to the manufacturer's instructions. The precise amounts of RNA used in each experiment are indicated in the corresponding figure legend.

Firefly and Renilla Luciferase activities were sequentially measured in a GloMax luminometer (Promega) using the Dual-Luciferase Reporter Assay System (Promega).

To count GFP-expressing foci, BHK-21 and A549 cells were transfected, and at 24 h, the culture media were removed and 30 μl of 1X PBS was added to the wells. The fluorescent spots were quantified using an ImmunoSpot Analyzer (Cellular Technology).

## Data analysis

Statistical analysis was performed using GraphPad Prism software (version 9.2).

## Data availability

All data are contained within the manuscript.

## Supporting information

This article contains Supporting information (10, 11, 20).

## Conflict of interest

The authors declare the following financial interests/personal relationships which may be considered as potential competing interests: Aspects of the molecules described herein are the subject of pending patent applications of GreenLight Biosciences. Laura A. Byk and Marcelo M. Samsa were employed by GreenLight Biosciences, Inc.
