# Supplementary material for: Improvement in the potency of a N1-methylpseudouridine-modified self-amplifying RNA through mutations in the RNA-dependent RNA polymerase
Source: J Biol Chem. 2025 Jul 17;301(8):110487. doi: 10.1016/j.jbc.2025.110487 (PMC12362096; doi:10.1016/j.jbc.2025.110487)
Supplement: Supplementary table 1 [file mmc1.pdf]

SUPPLEMENTARY TABLE 1

Restriction free cloning strategy for the plasmids used in this study. Nucleotide sequences of oligonucleotide primers and DNA templates for PCR are provided.

	First PCR		Second PCR	
	Primers	Template	Destination Plasmid	Description
VEEV-GFP	GLB 1<br>5'CTACGACATAGTCTAGTCCGCCAAGCTAGGCCACCATGGTGAGCAAGGGCGAG3'<br>GLB 2<br>5'CAGCTTGCCAATTGCTGCTGTATCATATGTTACTTGTACAGCTCGTCCATG3'	pEGFP-N1	TC-83	Insertion of GFP downstream of the subgenomic promoter
VEEV reporter replicon	GLB 3<br>5'TAGTCCGCCAAGCTAGGCCACCATGGAAGATGCCAAAAACATTAAGA3'<br>GLB 4<br>5'GCTCCTCGCCCTTGCTCACCATGGGCCCGGGGTTGCTCTC3'	piCRES_108_BspQI mut	VEEV-GFP	Insertion of firefly luciferase and FMVD 2A autoprotease upstream of GFP
	GLB 5<br>5'AGAACGCCTGAGCCGATCATCATCggcggcagcATGACTTCGAAAGTTTATGATCCAG3'<br>GLB 6<br>5'AGCAAACTTATGCTATCCTCTTCTTCCTCTTCgctgccgccTTGTTCATTTTTGAGAACTCGCT3'	pRL (Promega)	VEEV-GFP	Renilla luciferase inserted in nsp3 (ORF1)
VEEV reporter replicon dnsp4	GLB 19<br>5'CCGGATCACCATGTGCAGCATTCATTGCCTCTAGAAATATCGT3'<br>GLB 20<br>5'ATTAATTTGTCCGATTTGACTCCTTTCACGATATTTCTAGAGGCAATGAATG3'	VEEV reporter replicon	VEEV reporter replicon	Mutation in the nsp4 RdRp catalytic site (GDD464-466ASR)

C482G	GLB 27<br>5’ATTAATGGCAGACAGGGGCGCCACCTGGTTGAATA3’<br>115 5’ TTTTTTTTTTTTTTTTTTTGAAATAT3’	VEEV reporter replicon	VEEV reporter replicon	Low fidelity nsp4 (20)
C482Y	GLB 25<br>5’ATTAATGGCAGACAGGTATGCCACCTGGTTGAATA3’<br>115 5’ TTTTTTTTTTTTTTTTTTTGAAATAT3’	VEEV reporter replicon	VEEV reporter replicon	High fidelity nsp4 (10)
K290R	GLB 74 5’GTAATGGACTTAAGGAGAGACG3’<br>115 5’ TTTTTTTTTTTTTTTTTTTGAAATAT3’	VEEV reporter replicon	VEEV reporter replicon	Associated to favipiravir resistance (11)

10. L.L. Coffey, Y. Beeharry, A.V. Bordería, H. Blanc, and M. Vignuzzi, Arbovirus high fidelity variant loses fitness in mosquitoes and mice. *Proc. Natl. Acad. Sci. U. S. A.* **108**, 2011, 16038–16043
11. L. Delang, N. Segura Guerrero, A. Tas, G. Quérat, B. Pastorino, M. Froeyen et al. Mutations in the chikungunya virus non-structural proteins cause resistance to favipiravir (T-705), a broad-spectrum antiviral. *J. Antimicrob. Chemother.* **69**, 2014, 2770–2784
20. K. Rozen-Gagnon, K.A. Stapleford, V. Mongelli, H. Blanc, A.B. Failloux, M.C. Saleh et al. Alphavirus Mutator Variants Present Host-Specific Defects and Attenuation in Mammalian and Insect Models. *PLOS Pathog.* **10**, 2014, 1–15
